# Why do medical residents prefer paternalistic decision making? An interview study

**DOI:** 10.1186/s12909-022-03203-2

**Published:** 2022-03-08

**Authors:** Ellen M. Driever, Ivo M. Tolhuizen, Robbert J. Duvivier, Anne M. Stiggelbout, Paul L. P. Brand

**Affiliations:** 1 Department of Innovation and Research, Isala Hospital, Dokter van Heesweg 2, 8025 AB Zwolle, the Netherlands; 2 Lifelong Learning Education and Assessment Research Network (LEARN), University Medical Centre Groningen, Groningen, the Netherlands; 3 Faculty of Medical Science, University Medical Centre of Groningen, Groningen, the Netherlands; 4 Centre for Education Development and Research in Health Professions (CEDAR), University Medical Centre Groningen, Groningen, the Netherlands; 5 Parnassia Psychiatric Institute, The Hague, the Netherlands; 6 Department of Biomedical Data Sciences, Medical Decision Making, Leiden University Medical Centre, Leiden, the Netherlands; 7 Department of Medical Education and Faculty Development, Isala Hospital, Zwolle, the Netherlands

**Keywords:** Shared decision making, Evidence based medicine, Paternalism, Communication skills, Medical residents, Hidden curriculum

## Abstract

**Background:**

Although shared decision making is championed as the preferred model for patient care by patient organizations, researchers and medical professionals, its application in daily practice remains limited. We previously showed that residents more often prefer paternalistic decision making than their supervisors. Because both the views of residents on the decision-making process in medical consultations and the reasons for their ‘paternalism preference’ are unknown, this study explored residents’ views on the decision-making process in medical encounters and the factors affecting it.

**Methods:**

We interviewed 12 residents from various specialties at a large Dutch teaching hospital in 2019–2020, exploring how they involved patients in decisions. All participating residents provided written informed consent. Data analysis occurred concurrently with data collection in an iterative process informing adaptations to the interview topic guide when deemed necessary. Constant comparative analysis was used to develop themes. We ceased data collection when information sufficiency was achieved.

**Results:**

Participants described how active engagement of patients in discussing options and decision making was influenced by contextual factors (patient characteristics, logistical factors such as available time, and supervisors’ recommendations) and by limitations in their medical and shared decision-making knowledge. The residents’ decision-making behavior appeared strongly affected by their conviction that they are responsible for arriving at the correct diagnosis and providing the best evidence-based treatment. They described shared decision making as the process of patients consenting with physician-recommended treatment or patients choosing their preferred option when no best evidence-based option was available.

**Conclusions:**

Residents’ decision making appears to be affected by contextual factors, their medical knowledge, their knowledge about SDM, and by their beliefs and convictions about their professional responsibilities as a doctor, ensuring that patients receive the best possible evidence-based treatment. They confuse SDM with acquiring informed consent with the physician’s treatment recommendations and with letting patients decide which treatment they prefer in case no evidence based guideline recommendation is available. Teaching SDM to residents should not only include skills training, but also target residents’ perceptions and convictions regarding their role in the decision-making process in consultations.

## Background

In the Western world, shared decision making (SDM) is increasingly championed as the preferred model for patient care both by patients [1], clinicians [2, 3], healthcare organizations and policy makers [4, 5]. SDM is the process in which patients and clinicians collaborate to make a health decision that suits the individual patient’s medical situation, context, views and preferences [6]. There are several models that describe SDM. For example, Stiggelbout and colleagues distinguish four steps: informing the patient that a decision is to be made and that patient’s opinion is important, explaining options with pros and cons, exploring patients’ preferences and discussing patients’ decision role preference and make (or defer) the decision [7]. SDM is often contrasted with paternalistic decision making, whereby clinicians take the diagnostic or treatment action which they consider best for their patients, and ask for their consent [8]. Accumulating evidence suggests that patients who are more actively involved in decision making are more satisfied with the decision-making process and the decision itself, are more likely to adhere to treatment recommendations, and achieve better health outcomes [9–11]. Despite these advantages of SDM, its application in daily clinical practice remains limited [7, 12, 13]. A possible explanation is that not all health care professionals know what SDM is and how to apply it in practice [2–4]. They see SDM mainly as discussing the options with their advantages and disadvantages [3]. Physicians are increasingly trained and expected to base the information about the pros and cons of the different available options on evidence-based guidelines. Guidelines tend to overemphasize algorithmic rules and relatively strong recommendations based on limited or weak evidence [14], leaving relatively little room for including the patient’s preferences and values to arrive at a decision that fits the individual patient’s specific situation best [14–16].

Recently, we conducted a cross-sectional survey among 394 Dutch clinicians at different levels of seniority of their perceptions towards decision making in their medical encounters. This study showed that most clinicians preferred SDM, but in daily practice performed paternalistic decision making more often than they wanted. Unexpectedly, residents were considerably more likely than medical specialists to state to prefer and perform paternalistic decision making [3]. The reasons for their ‘paternalism preference’ remain unclear. It has been shown that residents and junior doctors feel uncertain about applying SDM in their encounters with patients [17–19], which may contribute to their preference for paternalistic decision making. To our knowledge the relationship between residents’ uncertainty and their decision-making behavior in medical encounters has not been studied to date. The overall positive attitude that physicians express towards SDM [2, 3], may be distorted by their belief that they already involve patients in decisions about their care [2–4]. It is unknown to what extent this also applies to residents. A thorough understanding of residents’ perception towards decision making is needed, because this generation of medical doctors will be instrumental in the further implementation of SDM as desired by patients [1], clinicians [2, 3], healthcare organizations and policy makers [4, 5]. More in-depth knowledge of residents’ views on the process of decision making in medical encounters and the factors affecting it, could help in designing SDM training programs to contribute to optimal patient centered care. This study was designed to develop a theoretical framework on how residents view the decision-making process in consultations with patients, and how they reflect on their own decision-making behavior in such encounters.

## Methods

### Study design

We used a constructivist grounded theory approach – a useful methodology for exploring processes and perceptions – to explore residents’ views on the decision-making process and their decision-making behavior in medical consultations. Consistent with grounded theory principles, we took an iterative approach: data collection and data analysis occurred simultaneously, with the ongoing data analysis informing the subsequent approach to data collection [20].

### Setting

In the Netherlands, residency programs last between five and 6 years. Residents work under direct or indirect supervision of medical specialists, and are increasingly autonomous in their practice over the years. Relevant for this study is that they see patients independently from their supervisors in outpatient clinics, on wards and at the emergency department. Residency programs consist of rotations at both academic and affiliated general teaching hospitals. We conducted our study at Isala Hospital, a large affiliated teaching hospital, offering rotations in 28 residencies. It serves as an affiliated hospital of the University Medical Center Groningen. The principles of evidence based medicine (EVM) and the importance of clinical practice guidelines are being taught in Dutch medical curricula, but SDM is not yet established as a core component of undergraduate and postgraduate medical education in the Netherlands.

### Participants

All medical residents who participated in our previous study [3], in which we assessed their preferred and usual decision-making roles with the modified Control Preference Scale (CPS), were invited via e-mail by the main researcher (EMD) to participate in this interview study. There was no working relationship or power relation between the researchers and the residents who were invited and we made it clear in the information about our research that participation was voluntary. Participants were not recruited based on specific characteristics. All participants were of Dutch nationality, none had a non-Western migrant background. There were six men and six women; seven participants were between 25 and 30 years of age, the other five between 30 and 35 years of age. Table 1 presents discipline, year of training, and preferred and usual decision-making role of participating residents (based on an earlier study) [3]. Residents who consented to participation in the study were invited consecutively until we had determined that our data were sufficient for answering our exploratory research questions (see Data analysis for more details).

**Table 1:** Participant characteristics (*N* = 12)

Characteristics of the medical residents		Number of residents
Specialty	Pulmonology	4
	Gastroenterology	3
	Emergency medicine	2
	Gynecology	1
	Urology	1
	Orthopedic surgery	1
Medical school	University of Groningen	8
	University of Amsterdam	2
	University of Rotterdam	1
	University of Nijmegen	1
Years of experience as junior doctor (range 6 months – 4.5 years)	0–1 year	2
	1–2 years	6
	> 2 years	4
Years of residency training (range 9 months – 6.0 years)	0–2 years	4
	2–4 years	6
	> 4 years	2
Decision making role preference as reported in previous study [3]	Paternalistic	4
	Informative	1
	Shared decision making	7
Usual decision making role as reported in previous study [3]	Paternalistic	7
	Informative	0
	Shared decision making	5

### Data collection

Two researchers (EMD and IMT), both trained in performing qualitative research interviews, conducted individual, semi-structured in-depth interviews with the participants between September 2019 and February 2020. Each interview was led by one researcher, with the other making field notes and asking additional questions if needed. EMD was acquainted with three participants because they were from the same year at the same university, those interviews were conducted by IMT. Each interview started by asking the residents to reflect on two recent clinical decisions that they had made in two medical encounters. We used these clinical scenarios as a basis for discussing the residents’ views on the process of medical decision making in their encounters, how they involved the patients in decisions, and what their preferred and regular behavior was in reaching a decision with or for the patient. We used an interview topic list to guide the flow of the interview. Interviews lasted between 40 and 60 min, were audio recorded and transcribed verbatim.

### Data analysis

Data analysis occurred concurrently with data collection in an iterative process informing adaptations to the interview topic guide when deemed necessary. Three researchers (EMD, IMT and PLPB) read, re-read and open coded each transcript independently. They met after the first two interviews and discussed discrepancies in the codes until they reached agreement about the initial coding list. Subsequent meetings between EMD, IMT and PLPB were conducted after every 2–3 interviews to discuss the coding. Codes were organized into related concepts, which were further developed into themes. Relations among themes were defined and discussed to arrive at a conceptual level of analysis, in consultation with the complete research team. We ceased data collection when sufficient information was achieved to reliably describe residents’ views on decision making and their behavior in medical encounters, without important gaps or leaps of logic [21]. We used Atlas.ti version 8.1 (Scientific Software Development GmbH, Berlin, Germany) to manage the data.

### Research team and reflexivity

In a constructivist grounded theory approach, concepts and themes arise through interaction with participants and other researchers in the team [20]. Therefore, it is important to take the background of the team members into account, because this influences data collection and interpretation [22]. The lead author (EMD) is a medical doctor with 1 year work experience as a resident in pediatrics before starting her PhD project on SDM. This experience as a resident was considered an advantage because it gave her an excellent insider understanding of the study subjects and their work contexts. IMT is a medical student who used this study as the subject of his master’s degree research thesis. He had experienced decision making in consultations mostly by observing residents and medical specialists doing this during consultations. RJD is a third year psychiatry resident with a background in educational research who also provided an insider perspective on the topic under study and contributed to relating developed theory with existing medical education research evidence. AMS is a professor of medical decision making with a background in epidemiology and a long track record of studying SDM in different settings. PLPB is a medical education researcher and professor, with a 20-year experience in pediatrics and research on treatment adherence and medical communication. The interdisciplinary research team allowed us to approach data analysis from various perspectives: those of medical student, resident, supervisor, and researcher of SDM and medical education, facilitating triangulation of the.

research findings. EMD, RJD and PLPB have experience in the application of SDM in their clinical work. After our previous study [3], we considered several potential explanations for the observed residents’ paternalism preference (e.g., resident uncertainty, time constraints, and the hierarchical nature of medicine in which residents are expected to follow supervisors’ guidance in making medical decisions), but the absence of scientific evidence to support any of these hypotheses prompted us to choose an explorative design for the current study. We therefore use a grounded theory approach to promote the development of a thorough account of the residents’ narratives of their decision-making views and behavior without an a priori model or hypothesis. Although all researchers had extensively studied SDM literature and view it as a useful model to promote patient involvement in medical decision making, they applied a number of safeguards against bias in analyzing and interpreting the data. In the interview guide, we deliberately used open-ended probing questions to encourage participants to discuss their own views on the decision-making process in medical encounters, and to describe their usual behavior in reaching decisions in such encounters, and avoided questions leading towards predefined SDM models. Being aware of researcher engagement and in striving to be as open as possible to the participants’ experiences and views, the interviewers tried to put aside their preconceptions about the phenomenon under investigation. During data analysis, the researchers kept an open mind for conflicting codes, reflecting on their interpretation of participants’ experiences and views, and resolving differences through discussion and consensus in an iterative process.

The study was carried out in accordance with the Declaration of Helsinki and was approved by The Ethical Review Board of the Dutch Association of Medical Education (NVMO) (file number 2019.5.6) and Isala Hospital’s Ethical Review Board (file number 200308). All participating residents provided written informed consent.

## Results

**Fig. 1:**
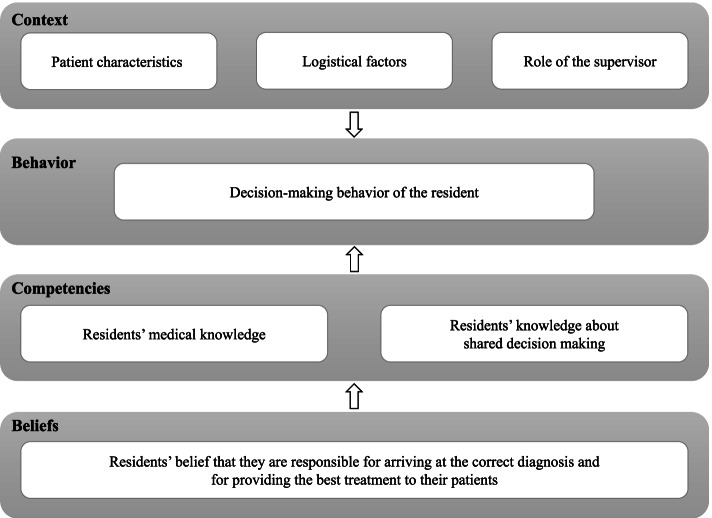
Factors influencing residents’ medical decision-making behavior. Legend: Participants described how active engagement of patients in discussing options and decision making was influenced by contextual factors (patient characteristics, logistical factors and supervisors’ recommendations) and by their medical and shared decision-making knowledge. The residents’ decision-making behavior appeared strongly affected by their belief that they are responsible for arriving at the correct diagnosis and providing the best evidence-based treatment

We identified four major themes that influenced residents’ views on and behavior in decision making: context, residents’ medical knowledge, residents’ knowledge about SDM, and residents’ beliefs about the doctor’s role in the decision-making process (see Fig. 1). We describe each theme in more detail, with representative quotes from the participants, including their discipline and their self-reported usual decision-making role from our previous study.

### Context

Residents described various contextual factors that influenced their decision-making behavior. We classified these contextual factors into three categories: patient characteristics, logistical factors and the role of the supervisor. All interviewed residents experienced lack of time and specific clinical settings, particularly acute situations, as barriers to involving the patient actively in the decision-making process.

*If someone comes into the emergency room with sepsis, then you need to, you need to just act and then you need to give them antibiotics. (P7, gastroenterology, SDM).*

*Umm.. yeah and if you admit a patient [..], then you still have options but then you just tell them “this is what we’re going to do”. Unless they object to it, then that is what you are actually going to do. (P4, pulmonology, paternalistic decision making).*

Some residents expressed reluctance to engage the patient in the decision-making process because they felt that this approach was not consistent with their supervisor’s work style. If a supervisor had proposed specific treatment for the patient in their discussion of the case with the resident, residents felt little room to discuss alternative treatment options and to involve the patient in the treatment decision, particularly during the first years of residency.

*That’s just my position as, as a resident. Umm, yeah.. if my supervisor wants me to (treat the patient as proposed by the supervisor), I’ll do it. (P8, emergency medicine, paternalistic decision making).*

### Residents’ medical knowledge

When residents thought that they lacked sufficient in-depth medical knowledge to be able to explain the advantages and disadvantages of different available options, they tended to propose a single treatment option that they were familiar with, and that they usually derived from national or local clinical practice guidelines. With increasing experience and more comprehensive medical knowledge, residents felt more confident to discuss different options with their patients and engage them in the decision-making process.

*For an appendectomy, I know what... what the odds are. But for antibiotic treatment, which I actually never really use, it hardly ever happens … .yeah, I don’t really know the stats that well. So in that sense I would steer towards surgery then. Because that’s something I know. (P8, emergency medicine, paternalistic decision making).*

### Limited knowledge about SDM

All interviewed residents had heard of the term SDM during medical school, for example in mandatory communication skills training, but none had followed specific SDM training. The residents’ descriptions of the decision-making process were consistent between participants, irrespective of their preferred decision-making role as reported in our previous study (Table 1) [3]. Residents described two approaches to decision making. In most cases, residents would propose a single specific treatment based on recommendations from evidence-based guidelines and would ask the patient to consent with this proposal. When no guideline recommendation was available to suggest a best treatment, they would inform the patient about benefits and risks of possible treatment options and let the patient decide. The residents called both these approaches SDM. They reported that they chose between these two approaches based on their assessment of the available evidence, the complexity of the decision, and the impact of the treatment on the patient. In a complex or severe clinical situation, residents would propose specific treatment more firmly to make sure that the patient received the treatment that residents considered best.

*Well, I think with complex issues it depends a bit on what kind of patient it is, but yeah, then you’re going to give them information where I think like, you really need me for. […*] *if it’s something that’s going to have a big impact on the patient’s life. Umm … while with a lipoma, you really don’t have that at all then, because then you just think, “you can decide, it really doesn’t make a difference to me”. (P2, urology, SDM).*

Residents related that they assessed the patient’s ability to make “the best decision” during the encounter, based on the patient’s social background and on their verbal and nonverbal responses to the questions and information provided by the residents. We asked all participants whether they ask their patients if and how they want to be involved in the decision making process. All participating residents stated that they never directly ask their patients this.

*Just based on a feeling, you can always tell. One patient wants to know all the ins and outs and they, well, then it’s obvious that they want to make the decision themselves. Some people will literally just say like “you’re the doctor, you just decide”. Well OK then, then you don’t really need to ask them. (P8, emergency medicine, paternalistic decision making).*

### Beliefs about the doctor’s role in decision making

All interviewed residents expressed the firm belief that they are responsible to arrive at the correct diagnosis and to provide the best treatment to their patients. They felt that this was their responsibility as a doctor.

*Look, as a doctor, of course you always want to do what’s best for the patient. (P10, pulmonology, paternalistic decision making).*

*You always want to do the best thing, but we always assume that what we think is best, is also best for the patient. (P1, pulmonology, SDM).*

The residents based this belief on the conviction that there was a correct diagnosis waiting to be uncovered for each patient, and that identifying this correct diagnosis would then logically lead to the best possible treatment option, based on the state of science or on evidence-based guidelines. They perceived the patient as a passive partner in this process, not as a co-creator of the choice that would best suit the patient’s needs and preferences.

*If a patient comes in with pneumonia, then you’re obviously not going to ask them which antibiotic they want. That’s, that’s just a decision based on protocol. That’s, that’s not a choice. (P10, pulmonology, paternalistic decision making).*

*Interviewer: “how do you know what the best option is?” Resident: “It’s been supported by scientific research”. (P7, gastroenterology, SDM).*

In order to achieve the best results for their patients, residents would steer the decision-making process towards their preferred option, especially when they were convinced that there was evidence to clearly prefer one treatment option.

*I didn’t leave her a choice at all, no. I just thought: previously healthy woman, this is what we’re going to do. (P9, gastroenterology, SDM).*

*Umm … how do we achieve that I actually get my way? Because I really do want him to have the best recovery. (P6, emergency medicine, paternalistic decision making).*

*Suppose umm … option A is clearly better than option B, then I’ll do my best to get that across to the patient. […] and I feel that that’s just normal, I mean, you became a doctor so that you can cure someone and uh … then you want to do that in the way that has the best results. (P8, emergency medicine, paternalistic decision making).*

## Discussion

In residents’ narratives about their decision-making behavior in medical encounters, we identified four themes that help to understand how residents think about the decision-making process in medical consultation. These themes were context factors, residents’ medical knowledge, residents’ SDM knowledge and skills and residents’ beliefs about the doctor’s role in decision making.

When reflecting on factors influencing their decision making, residents first mentioned a number of context factors that have been described previously as barriers to apply SDM in clinical practice: patient characteristics [2, 23], time pressure [2, 24], clinical setting (e.g. emergency situation) [23, 25], and the role of the supervisor [23, 26]. They also reported that their lack of medical knowledge and their lack of SDM knowledge and skills hindered involving patient in the decision-making process, in accordance with previous studies [4, 23, 27], Similar contextual and competency barriers have been described previously in learning and practicing other complex skills, in health care communication [28, 29], practicing evidence based medicine [30, 31], and in surgical procedures [32]. In addition to these earlier reported barriers, however, the residents in our study also expressed a strong inner conviction that they are responsible for making the correct diagnosis and providing the best treatment for their patients. They felt that this was their responsibility as a doctor. This conviction appeared in the interviews with all participants, irrespective of the decision-making model preference they expressed in our previous study [3]. When residents talked about the “best” treatment, they invariably based this on recommendations from evidence-based guidelines. It has been argued that good care involves the integration of EBM and patient centered communication (PCC) skills to make the best decisions for individual patients [16, 33, 34]. Although the founding fathers of the EBM movement emphasized that evidence-based practice implies the integration of the best available medical evidence, the physician’s expertise and the patient’s views and preferences [35], the guidelines are commonly presented to medical students and junior doctors as the “single best answer” and the “correct thing to do” [14, 36, 37], which discourages physicians from making decisions tailored to the individual patient’s context, views and preferences [14, 16, 33].

The principle of SDM is rooted in the conviction that the patient’s specific context, views or preferences may be valid reasons to deviate from guideline recommendations and choose another treatment option that fits the patient’s situation best [7]. The residents in our study did not express awareness of that perspective on reaching a decision tailored to the patient’s own expressed views and preferences, but considered it their responsibility to decide for their patients which treatment would be best for them. That these strong beliefs on professional tasks and responsibilities have a major impact on the residents’ decision-making behavior in clinical encounters reflects findings from a recent study in which the development of professional identity in medical students was analyzed using the so-called levels of change model [38]. This model is an adaptation of Bateson’s theory of the six ‘logical levels’ at which people think, learn, change and function: environment, behavior, competencies, beliefs, identity, and mission [39]. The interviewed residents in this study described how the context *(environment)* and their knowledge and skills *(competencies)* affected their decision making (*behavior*). They related how their decision making was guided by their convictions about their role as a doctor in the decision-making process *(beliefs*). The strength of their convictions and the firmness with which they were expressed suggested to us that these convictions could be part of their professional *identity* as a doctor [40–42]. Residents not only expressed the conviction that they are responsible for providing the best treatment in each patient, they also expressed the positivist view that each patient has a *correct diagnosis* which needs to be uncovered to allow them to provide patients with the best evidence-based treatment. They did not perceive the patient as a partner in this process, co-creating a treatment that would best suit the patient’s needs and preferences [8]. This attitude reflects the ongoing dominance of the positivist paradigm in medical science [43]. A recent analysis of medical education materials showed that physicians are typically presented as the ones responsible for making the decision, and for convincing patients to follow their plans [44]. Thus, there appears to be a hidden curriculum in medical education steering medical students and residents away from SDM towards a more paternalistic model in their medical encounters. This helps to understand the findings of our previous study, in which most residents preferred paternalistic decision making [3]. The high status placed on scientific evidence in guiding medical decisions may lead to “cookbook medicine”*,* thereby reinforcing to medical students and residents that there is only one way of dealing with a given clinical situation [16, 45, 46]. We propose that this deeply rooted positivist paradigm in medical science may help to explain the relatively small effects of training physicians in SDM skills on their decision-making behavior in subsequent consultations [47–51] or their adoption of the patient perspective [52]. It raises the question of how this positivist professional identity is being shaped in graduate and postgraduate medical education programs, and how an appreciation of the uniqueness of each patient’s context, views and preferences can be fostered throughout the medical education continuum.

The main limitations of our study include the single-institution context and the relatively small number of interviewed residents. Although we have no a priori reason to believe that residents from other hospitals have different beliefs about the role of a doctor in the decision-making process with their patients, further studies are needed to triangulate our findings across different settings (e.g. primary care) and countries. Although the participation in our previous study may have affected residents’ views on SDM, the remarkably similar responses on decision making in clinical encounters and the factors affecting this between participants who expressed preference for different decision making models in our previous study argues against responder bias.

Our results suggest that training residents in the complex skills of SDM should address the residents’ convictions and beliefs regarding their professional tasks and responsibilities in the decision-making process [39, 53, 54]. Even if we succeed in shaping medical students’ and residents’ professional identity towards a model embracing SDM, the role model behavior that their supervisors express during the clinical phases of medical education is likely to have a strong effect on residents’ decision-making behavior in daily practice [55]. Successfully training students and residents in SDM should therefore include training the supervisors, to reduce the role models’ effects in the hidden curriculum. The relentless drive to improve the evidence base of clinical medicine should be combined with a firm commitment to patient centeredness [33], to promote medical learners to embrace SDM, both in undergraduate and postgraduate medical education.

## Conclusion

Residents’ paternalism preference in decision making appears to be affected by contextual factors, their medical knowledge, and their knowledge about SDM, but most strongly by their beliefs and convictions about their professional responsibilities as a doctor. They feel that they are responsible for making the correct diagnosis and providing the best treatment for their patients, not with their patients. These results suggests that shared decision-making teaching in residency should not be limited to communication skills training, but should also target residents’ professional beliefs and values regarding their own and the patient’s role in the decision-making process. This may be required to enhance the implementation of SDM in daily clinical practice, as the pinnacle of evidence based practice.

## Data Availability

The datasets generated and/or analysed during the current study are not publicly available due the qualitative study design, in which the dataset consists only of the interview transcripts and the coding document, but this dataset is available from the corresponding author on reasonable request.

## Abbreviations

SDM: Shared decision making
EBM: Evidence based medicine
